# Effectiveness of a hybrid digital substance abuse prevention approach combining e-Learning and in-person class sessions

**DOI:** 10.3389/fdgth.2022.931276

**Published:** 2022-08-03

**Authors:** Kenneth W. Griffin, Christopher Williams, Caroline M. Botvin, Sandra Sousa, Gilbert J. Botvin

**Affiliations:** ^1^Department of Global and Community Health, George Mason University, Fairfax, VA, United States; ^2^National Health Promotion Associates, White Plains, NY, United States; ^3^Purchase College, State University of New York, Purchase, NY, United States; ^4^Teachers College, Columbia University, New York, NY, United States; ^5^Department of Population Health Sciences, Weill Cornell Medical College, New York, NY, United States

**Keywords:** substance abuse, adolescence, prevention, hybrid, e-learning

## Abstract

**Background:**

Effective school-based programs for preventing substance abuse offer considerable public health potential. Yet limited class time and uneven implementation fidelity can be barriers to widespread adoption and high-quality implementation. A hybrid digital approach may be effective and help address these barriers.

**Objective:**

To evaluate the effectiveness of a hybrid substance abuse prevention program for middle school students consisting of e-learning modules and in-person class sessions.

**Design:**

Twenty-three United States (U.S.) middle schools were randomly assigned either to an intervention condition (13 schools) or a treatment-as-usual control condition (10 schools) where standard health education material was delivered. There were 1,447 participants who completed the pre-test and post-test assessments, of which 48.3% were male and 51.7% female.

**Intervention:**

The hybrid digital intervention consisted of 14 brief e-learning modules and six classroom sessions adapted from an evidence-based program designed for classroom implementation to increase knowledge of adverse consequences of substance use and improve social skills, personal coping skills, and skills for resisting social influences to smoke, drink, or use drugs.

**Measures:**

Participating students completed online pre-test and post-test surveys to assess substance use, knowledge, and life skills.

**Results:**

There were significant reductions in substance use for the hybrid digital condition compared to the control condition as well as significant increases in health knowledge, skills knowledge, and life skills.

**Conclusions:**

A hybrid digital approach to substance abuse prevention is effective and offers potential for overcoming common barriers to widespread adoption and high-quality implementation.

## Introduction

Substance abuse continues to be an important public health problem that contributes greatly to morbidity and mortality rates throughout the United States (1) and globally (2). It is estimated that 90% of American adults who meet the clinical criteria for addiction started using one or more substances before the age of 18 (3). The prevalence rates of alcohol, tobacco, and other forms of substance use increase from early to late adolescence and peak during the transition to young adulthood, as shown in multiple large epidemiological studies in the US (4). The initiation of substance use during adolescence contributes not only to later use, abuse, and addiction (5–9), but it also increases the likelihood of a variety of later negative social, behavioral, and mental health problems (10). Programs that effectively prevent substance use during adolescence are critical for reducing the many documented negative individual and societal consequences of early onset of substance use and abuse.

### School-based prevention

There is a considerable literature of research testing the effectiveness of substance abuse prevention approaches for schools, families, and communities (11). A particular focus of this research has been on school-based programs to prevent the onset and early stages of substance use and other health risk behaviors. Schools are well-suited for substance abuse prevention efforts, particularly for universal or primary prevention approaches, since they provide relatively easy access to the general population of youth and play a major role in educating, socializing, and preparing students for future success (12).

Several meta-analyses and systematic reviews have examined the effectiveness of school-based programs to prevent alcohol, tobacco, and other forms of substance use (13–15). Although the methodologic rigor and theoretical foundation of prevention programs included in these reviews vary considerably, the findings are useful in identifying the characteristics of effective programs. These reviews not only show that school-based prevention can be effective, they also show that the most effective programs are based on relevant psychosocial theory, target salient risk and protective factors, focus on building skills in drug resistance and general competence skills, and are delivered by teachers or other program providers using interactive teaching methods (11).

As research identifying effective school-based prevention programs has accumulated, there has been a concomitant increase in efforts to move from science to practice by promoting the use of evidence-based approaches. These efforts have been prompted by the recognition that unless there is widespread adoption and implementation of prevention programs that have been rigorously tested and proven effective, the promise of these programs is unlikely to be realized. However, efforts to promote the adoption and implementation of evidence-based prevention programs in schools have had only limited success, with most schools continuing to use untested, unproven, or ineffective prevention approaches (11, 16, 17).

### Overcoming adoption and implementation barriers

Notwithstanding the demonstrated effectiveness of evidence-based prevention programs delivered by teachers or other program providers in school settings, barriers to adoption and implementation provide compelling reasons for considering the use of other delivery modalities. Two common barriers to the widespread adoption and effective implementation of evidence-based prevention programs are (1) limitations on classroom instructional time and (2) uneven implementation fidelity by program providers (18). The delivery of prevention content through an online or e-learning format may help surmount these barriers and facilitate greater adoption and implementation of evidence-based prevention programs in school settings. Moving some portion of the content of school-based substance abuse prevention programs to an e-learning format can reduce the burden on teachers and the amount of time needed for in-class instruction.

Furthermore, research has shown that implementation fidelity is highly variable when programs are delivered by teachers or other program providers in schools and other real-world settings, and that poor implementation reduces intervention effectiveness (19, 20). Teachers and other program providers may not implement evidence-based prevention programs in their entirety, may adapt interventions in ways that diminish the core components of the original program design, or may fail to use interactive skills training methods in their instructional approach (18, 21).

### Potential of digital health interventions

A growing body of research conducted with target audiences of all ages and backgrounds provides evidence of the utility, appeal, efficacy, and cost-effectiveness of digital health interventions for health and wellness, personal behavior change, and other behavioral, psychological, and vocational educational purposes (22–26). Digital health interventions allow for the transfer of health information in a standardized, flexible, accessible, convenient, and direct manner that is less costly because direct staff involvement is minimal. They can provide media-rich, entertaining, and pedagogically innovative content that leads to improvements in knowledge acquisition as well as student satisfaction and motivation (27–29).

Digital health intervention approaches may be particularly appropriate for adolescents who actively consume digital content, are comfortable and familiar with digital devices, and are the largest single group of internet users, with over 95% accessing the internet daily (30). A recent meta-analysis identified 36 studies examining the impact of digital health interventions on a variety of health behaviors among youth including physical activity, dietary change, weight loss, and chronic disease self-management and found a significant aggregate effect size across studies (31). A systematic review and meta-analysis of digital health interventions provided to adolescents in school settings found significant effects of these interventions on physical activity, screen time, and fruit and vegetable intake (32). Regarding the prevention of adolescent alcohol and other drug use, a systematic review of universal digital health preventive interventions found that six of the nine interventions identified produced statistically significant but modest effects for alcohol and/or other drug use outcomes (22). Although this research is promising, further research is warranted to identify effective digital health intervention approaches for adolescent substance abuse prevention.

### Blending digital and in-class modalities

One limitation of prevention programming provided entirely in an online or digital format is that it may limit the use of teaching strategies for prevention with demonstrated effectiveness, such as facilitated group discussion and behavioral skills training. Arguably, these interactive teaching methods may be best suited for in-class implementation by a trained provider. Therefore, a prevention model that uses a hybrid or blended learning approach could maintain the strengths of both digital health interventions and interactive classroom instruction. In this context, a hybrid or blended delivery model refers to one in which both an in-person and digital delivery modality are used to teach prevention material.

One potential hybrid model for prevention mirrors the “flipped” or “inverted” classroom where didactic content traditionally delivered in classrooms is provided digitally and classroom time is reserved for more interactive learning activities (33). In the context of a prevention program, this would involve presenting didactic content in online e-learning modules followed by classroom time with instructor-student and student-student discussion and interactions that facilitate pro-health norm-setting and skills-training through behavioral rehearsal. Such a hybrid delivery model offers the potential for increasing the feasibility and fidelity of evidence-based prevention by providing content in a digital format using technologies familiar to students and educators. At the same time, a hybrid delivery approach would maintain the benefits of interactive class sessions by providing opportunities for norm-setting and skills-training activities.

### Life skills training

A leading evidence-based substance abuse prevention approach is *Life Skills Training* (LST), a universal prevention program designed to be implemented with all students in a regular classroom setting. In addition to teaching information about the adverse health and social consequences of substance abuse, LST teaches personal self-management skills, social skills, and other life skills needed to resist social influences to engage in substance use and to acquire adaptive coping skills, develop healthy relationships, and increase resilience (34).

LST has been extensively tested in a series of randomized controlled trials reported in over 35 peer-reviewed publications involving 18 separate cohorts (16). This program has been proven effective when taught by teachers, peer leaders, and prevention specialists using a combination of didactic instruction, small-group discussion, and cognitive-behavioral skills-training activities. These studies showed reductions of 50% or more relative to controls in cigarette smoking, alcohol use, marijuana use, and use of illicit drugs among students receiving the LST program, as well as improvements in risk and protective factors associated with adolescent substance abuse. LST also has been shown to reduce violence, aggression, and delinquency among middle school youth (35). Long-term follow-up data collected from students who participated in the LST program in middle school have shown that program effects lasted well into young adulthood.^1^ Follow-up studies also have shown effects on behaviors not directly addressed by LST including risky driving (36), HIV risk behavior among young adults (37), and methamphetamine use (38). Taken together, findings from this body of research indicate that LST, when implemented in class by teachers or other program providers, can produce both immediate and long-term prevention effects that last into young adulthood, and that these effects can generalize to other risk behaviors not specifically addressed in the program.

### Goal of the present study

The present study was designed to test the effectiveness of a hybrid digital program adapted from the LST substance abuse prevention program. The main hypothesis guiding this study is that the hybrid program would produce significant reductions in substance use relative to controls. It was also hypothesized that the hybrid digital program would produce significant improvements in health knowledge concerning the adverse effects of substance use, skills knowledge, and life skills. If effective, this approach would offer the potential of overcoming common barriers to widespread adoption and quality implementation of evidence-based prevention programs by decreasing the amount of class time required and reducing concerns about implementation fidelity.

## Materials and methods

Using national lists of middle school principals, teachers, and district-level administrators, schools were randomly selected from different geographic areas and emailed recruitment packets with a description of the study. Schools that agreed to participate in the study were matched by geographic region and enrollment size, and then randomized to either intervention or control conditions. The final roster of schools enrolled in the study represented a diverse set of schools from different regions of the US, including the Northeast (21.7%), Midwest (21.7%), South (39.1%) and the Western states (17.3%). Participating schools were mostly middle schools with students (ages 11–14) in grades 6–8 (58%) or grades 6–9 (12%), along with schools serving students from kindergarten to grade 8 (12%) or kindergarten to grade 12 (18%). Data in the present study were collected from the Fall of 2018 through the Spring of 2020.

### Sample

A total of 1,799 students from 23 middle schools voluntarily participated in the current study and completed the pre-test survey. Of these, 80.4% (*N* = 1,447) completed the post-test survey and comprised the analysis sample for examining intervention effects. This sample was 48.3% male and 51.7% female. The racial makeup of the sample was White (67.4%), Black (16.8%), American Indian or Alaskan Native (4.1%), Asian (2.8%), Native Hawaiian and Other Pacific Islander (1.0%). About 12.2% of participants reported that they were Latino/Hispanic. The mean age of participants was 11.94 years old (SD = 0.86) and most participating students were in the 7th grade (48%) or 6th grade (38%).

### Research design

The effectiveness of the intervention was assessed through a cluster-randomized control-group design where schools were matched by geographical region and enrollment size prior to randomization. Schools within each block were then randomly assigned to either receive the intervention (13 schools) or serve in the control group (10 schools). Students in the intervention schools (*n* = 755) received a substance abuse prevention program that consisted of e-learning modules and teacher-led class sessions, while students in the control schools (*n* = 1,044) received the standard health education curriculum provided by their schools.

### Procedure

Study participants completed an online pre-test survey prior to the intervention and a post-test survey ~4 weeks after completion of the intervention. The surveys assessed self-reported substance use behavior, demographic information, knowledge of the adverse health and social effects of substance use, skills knowledge, and life skills. Unique identification codes were used to link pre-test and post-test surveys. To preserve confidentiality, student names were not included on any data collection materials. Students completed pre-tests and post-tests using computers, laptops, or other portable devices during a regular classroom period or computer lab session. All study procedures were similar to those used in previous prevention studies (39, 40) and were approved by an Institutional Review Board. A referral protocol that provided a free and confidential 24-h hotline was available for any students who might experience emotional distress. No participating students reported distress and the protocol was not activated.

### Intervention

The content for the e-learning program was adapted from the *Life Skills Training* (LST) program in a careful iterative process to ensure integrity to the LST model. The adapted intervention tested in this study was designed to be appropriate for diverse populations. The language and imagery used in the written and video materials, as well as the video hosts and animated characters used in the hybrid program materials, were designed to be representative of diverse cultures and backgrounds in order to increase universal appeal. In addition, focus groups were conducted with middle school students, teachers, and parents from diverse geographic (urban, suburban, and rural) and socioeconomic backgrounds to obtain feedback on the content, feasibility, relevance, usability, and appeal of the program materials. This feedback was then incorporated into revisions as the materials were developed and finalized. The final intervention consisted of 14 e-learning modules and six in-person class sessions. The e-learning modules ranged from 4 to 10 min in length (M = 6.43 min) and the six classroom sessions were each ~40 min long.

The e-learning modules were designed to provide the program content, whereas the in-person class sessions were intended for small-group discussion and to provide students with an opportunity to practice the skills taught online. The class materials consisted of: (1) a comprehensive, step-by-step Teacher Manual with session goals, objectives, content, interactive activities, and instructions for implementing the program; and (2) a set of review slides containing the information and skills covered in the online program and used to guide students during the class sessions.

#### Program sequence

The program was organized into three conceptually linked content blocks, as shown in Table 1. Students viewed a set of online e-learning modules prior to attending the relevant classroom session focused on facilitated skills practice for those topics. The number of classroom sessions per block varied, such that more sensitive topics (i.e., substance use) incorporated more classroom sessions. In order to provide a real-world test of the intervention, teachers were permitted flexibility in how they implemented the program. Some teachers opted to implement the online program and classroom sessions so that: (1) students viewed the e-learning modules as homework and the teacher then delivered the live classroom sessions corresponding to those modules; (2) students viewed the e-learning modules at school in a computer lab and the teacher then delivered the corresponding live classroom sessions; or (3) students viewed portions of the e-learning modules during class time on computers or individual digital devices, and the teacher delivered the corresponding live classroom sessions until all digital and live content had been implemented. Sites required 6–12 weeks to complete the full set of e-learning modules and in-person classroom sessions, which fits into the duration of most academic terms.

**Table 1:** Program sequence for the adapted hybrid LST program.

**Block 1: Five e-learning modules and one classroom session** • e-Learning modules: Introduction, self-image & self-improvement, making decisions, advertising, violence and the media (length: 28 min) • Classroom session 1: Self-image, goal-setting, making decisions (activity time: 30–40 min)
**Block 2: Four e-learning modules and two classroom sessions** • e-Learning modules: Coping with anxiety, coping with anger, communication skills, social skills (length: 26 min) • Classroom session 2: Coping with stress, anxiety and anger (activity time: 30–40 min) • Classroom session 3: Social relationship skills (activity time: 30–40 min)
**Block 3: Six e-learning modules and three classroom sessions** • e-Learning modules: Assertiveness, resolving conflicts, smoking, alcohol, marijuana, prescription drug misuse (length: 39 min) • Classroom session 4: Assertiveness and resolving conflicts (activity time: 30–40 min) • Classroom session 5: Resisting tobacco, alcohol, and marijuana use (activity time: 30–40 min) • Classroom session 6: Prescription drug misuse (activity time: 30–40 min)

#### e-Learning modules

As shown in Table 2, the e-learning modules included an Introduction to the program followed by modules focused on: (1) Self-Image & Self-Improvement, (2) Making Decisions, (3) Advertising, (4) Violence and the Media, (5) Coping with Anxiety, (6) Coping with Anger, (7) Communication Skills, (8) Social Skills, (9) Assertiveness, (10) Resolving Conflicts, (11) Smoking and e-Cigarette Use, (12) Alcohol, (13) Marijuana, and (14) Prescription Drug Misuse.

**Table 2:** Description of online e-learning modules.

**e-Learning module (seat time)**	**Lesson goals**	**Key skills**
Introduction (3 min)	To welcome and introduce the Life Skills Training Program.	Navigating the online course.
Self-image and self-improvement (7 min)	To teach what self-image is, how it is formed, how it relates to behavior, and how it may be improved.	Self-analysis, self-improvement, goal-setting, reframing thoughts.
Making decisions (7 min)	To teach how to make decisions and solve problems independently.	Decision analysis; 3 C's of effective decision-making (Clarify, Consider, Choose); resisting group pressure.
Advertising (6 min)	To increase awareness of the techniques employed by advertisers to manipulate consumer behavior and how to resist these techniques.	Analyzing ads; recognizing techniques; separating fact from fiction, want from needs.
Violence and the media (5 min)	To increase awareness of how the media influences perceptions about violence and how to check media presentations against realities.	Analyzing perceptions about violence; separating fact from fiction; resistance to media distortions.
Coping with anxiety (7 min)	To teach what anxiety is, common situations which cause it, and techniques for coping with anxiety.	Recognizing anxiety and its physical effects; learning healthy techniques to deal with anxiety; progressive relaxation; mental rehearsal/visualization; breathing.
Coping with anger (4 min)	To teach anger recognition and common situations which cause it, and techniques for coping with anxiety.	Recognizing anger, its physical effects and multiple consequences; identifying reasons and learning techniques to control anger.
Communication skills (5 min)	To teach effective communication strategies.	Using verbal and non-verbal communication; techniques for avoiding misunderstandings;
		clarifying; asking questions; being specific; paraphrasing.
Social skills (10 min)	To teach social skills for developing and maintaining successful interpersonal relationships. Teach skills pertaining to close personal relationships, interaction with others, and planning social activities.	Making social contacts; giving and receiving compliments and other feedback; conversation skills, making plans; having self-awareness.
Assertiveness (8 min)	To teach the differences in being aggressive and assertive and how to become more assertive and resist peer pressure to use drugs.	Reflecting on actions taken, consequences; repertoire of refusal responses; verbal and non-verbal assertiveness; self-respect.
Resolving conflicts (5 min)	To teach strategies for resolving conflicts and review previous skills as they relate to conflict resolution.	Analyzing conflict resolution choices; controlling anger; building consensus; problem solving; negotiation and compromise.
Smoking and e-cigarette use (8 min)	To teach information about cigarette smoking and e-cigarette use and to counter common myths and misconceptions.	Analyzing data; checking assumptions; considering pros/cons; separating fact from fiction.
Alcohol (6 min)	To teach information about alcohol use and to counter common myths and misconceptions.	Analyzing data; checking assumptions; considering pros/cons; separating fact and fiction.
Marijuana (5 min)	To teach information about marijuana use and to counter common myths and misconceptions.	Analyzing data; checking assumptions; considering pros/cons; separating fact from fiction.
Prescription drug misuse (7 min)	To teach information about prescription drug misuse, to counter common myths and misconceptions, and to recognize and resist negative peer pressure.	Analyzing data; checking assumptions; considering pros/cons; separating fact from fiction; recognizing persuasive tactics and assertively refusing.

Each e-learning module began with a brief videotaped segment that included a male and female middle school student who served as co-hosts and introduced the content to be presented. Each module also included visual storytelling using animated characters, and a series of interactive knowledge checks were used throughout the e-learning program to assess knowledge acquisition. Once students chose their answer, they were given feedback on their choice and learned of potential outcomes. Finally, the two video hosts concluded the module.

#### Class sessions

As shown in Table 3, the six classroom sessions focused on: (1) Self-Image, Goal-Setting, and Making Decisions; (2) Coping with Stress, Anxiety, and Anger; (3) Social Relationship Skills; (4) Assertiveness and Resolving Conflicts; (5) Resisting Tobacco, Alcohol, and Marijuana Use; and (6) Prescription Drug Misuse. Each class session was designed to complement the corresponding e-learning module and review and elaborate on information learned during the e-learning sessions. For example, the classroom session on Prescription Drug Misuse briefly reviewed key definitions and topics introduced in the e-learning module before shifting to a series of discussion points, interactive activities, and opportunities for skills practice through behavioral rehearsal. During the class session, students discussed reasons why people do and do not abuse prescription drugs and brainstormed their own reasons for not abusing them. Students worked together to generate examples of what it means to abuse prescription drugs, and created rules for their safe use. The classroom lesson provided behavioral rehearsal scenarios focusing on refusal skills in the context of prescription drug offers from peers. Students learned strategies to decline prescription drugs when offered or when asked to share their own prescription drugs. During this activity, teachers guided students as they practiced these skills and acted out realistic scenarios with their peers.

**Table 3:** Description of class sessions.

**Class session**	**Lesson goals**	**Key skills**
Self-image, goal-setting, making decisions	To introduce the program and review ground rules. To learn and practice goal-setting, self-improvement, and decision making.	Self-analysis, self-improvement, goal-setting. Decision analysis; 3Cs of effective decision-making (Clarify, Consider, Choose).
Coping with stress, anxiety and anger	To learn to recognize symptoms of stress and anxiety. To give students tools to effectively manage and control stress and anxiety.	Recognizing anxiety and its physical effects; common situations that cause stress, anxiety, and anger; easy and healthy techniques to cope with stress, anxiety, and anger.
Social relationship skills	To review and practice using verbal and nonverbal communication skills to avoid misunderstandings and arrange for social activities.	Verbal and non-verbal communication; avoid misunderstandings; start, continue, and end a conversation; invite others to social activities; respond to invitations to social activities.
Assertiveness and resolving conflicts	To review and practice assertive skills and conflict resolution strategies.	Verbal and non-verbal assertive skills; problem-solving and negotiation skills to resolve conflicts.
Resisting tobacco, alcohol, and marijuana use	To increase students' ability to resist pressure to smoke, drink, and use marijuana and illicit drugs using assertive skills.	Refusal skills techniques; resisting peer pressure; using refusal skills for saying “no.”
Prescription drug misuse	To increase students' ability to resist pressure to abuse or misuse prescription drugs, including opioids.	Prescription drugs; prescription drug and over-the-counter drug misuse; refusal techniques.

#### Online provider training workshop

Prior to the implementation of the intervention, program providers in the intervention schools completed an asynchronous online training workshop. The workshop was designed to train providers to effectively implement the intervention, including how to structure the delivery of the online e-learning modules and corresponding classroom sessions. In the 6-h self-paced training, they explored the conceptual model of the prevention program and its underlying theory, methods, and effectiveness. Participants reviewed the scope and sequence of the adapted hybrid program including the goals, objectives, and learning activities of the e-learning modules and classroom sessions. Participants learned and practiced the cognitive–behavioral skills training methods needed to successfully implement the classroom sessions of the program (e.g., facilitating and guiding classroom discussions, demonstrating new skills, coaching students through small-group behavioral rehearsal or skills practice, and providing positive feedback and reinforcement). Program providers were given instruction on practical implementation issues including classroom management strategies, how to deal with potential disclosure of sensitive information by students, and how to establish ground rules for the classroom sessions (e.g., all students should be given the opportunity to participate, everyone's contributions must be respected without criticism).

### Measures

Data were collected using an online self-report survey administered prior to the intervention and ~4 weeks after the completion of the intervention. Data collection for students in the control condition was on a similar schedule to ensure that the interval between the pre-test and post-test was similar for both conditions. The survey contained items that assessed student demographic information, substance use, health and skills knowledge, and life skills.

#### Demographic information

Data concerning the demographic characteristics of the participants were collected using standard survey items assessing gender, age, and race/ethnicity.

#### Substance use

Substance use items assessed the frequency of smoking, e-cigarette use, alcohol use, drunkenness, and marijuana use on a nine-point Likert scale. Response options included “Never” (1), “A few times but NOT in the past year” (2), “A few times a year” (3), “Once a month” (4), “A few times a month” (5), “Once a week” (6), “A few times a week” (7), “Once a day” (8), and “More than once a day” (9). Examples include: “About how often (if ever) do you smoke marijuana (weed, pot)?” and “About how often (if ever) do you drink beer, wine, or hard liquor (excluding religious ceremonies)?” Students were also asked about how often they share prescription medications with others, using the same response options. The substance use items are similar to those used in national surveys in the United States such as the *Monitoring the Future* study funded by the National Institute on Drug Abuse (41) and have been used in previous studies testing preventive interventions (16).

#### Health knowledge

Sixteen “True/False” items used in previous prevention research (42) assessed health knowledge. Health knowledge items were used to assess student understanding of health and social issues associated with substance use. There were five items that assessed Smoking and e-Cigarette Use Knowledge (e.g., “Smoking a cigarette causes your heart to beat slower”); five items that assessed Alcohol Use Knowledge (e.g., “A serving of beer or wine contains less alcohol than a serving of liquor”); three items that assessed Marijuana Use Knowledge (e.g., “Smoking marijuana causes your heart to beat faster”); and three items that assessed Prescription Drug Misuse Knowledge (e.g., “Prescription drugs are safe as long as a doctor prescribes them”). An overall Health Knowledge Score was calculated that reflected the total number of the 16 items answered correctly.

#### Skills knowledge

Twenty-seven “True/False” items used in previous prevention research assessed Skills Knowledge (42). Knowledge items assessed key intervention skills including Decision-Making, Coping with Anxiety and Anger, Communication and Social Skills, Assertiveness, and Conflict Resolution. Additional knowledge items included material on Self-Image, Advertising/Media, and Violence and the Media. There were three items that assessed Self-Image Knowledge (e.g., “Self-image is linked to our experience with specific situations”); three items that assessed Decision-Making Knowledge (e.g., “Decisions you make are influenced by those around you”); three items that assessed Advertising/Media Knowledge (e.g., “Companies advertise because they want you to have all the facts about a product”); three items that assessed Violence & the Media Knowledge (e.g., “Most video games contain violence”); three items that assessed Coping with Anxiety Knowledge (e.g., “There is very little you can do when you feel anxious”); three items that assessed Coping with Anger Knowledge (e.g., “Letting anger get out of control can escalate a conflict”); three items that assessed Communication Skills Knowledge (e.g., “Paraphrasing can clear up misunderstandings”); three items that assessed Social Skills Knowledge (e.g., “Relaxation techniques are not helpful when meeting new people”); three items that assessed Assertiveness Knowledge (e.g., “Saying how you feel is a good way to be assertive”); and three items that assessed Conflict Resolution Knowledge (e.g., “It is best to solve conflicts by giving in to what other people want”). An Overall Skills Knowledge score was calculated based on the total number of the 30 items answered correctly.

#### Life skills

Twenty items with established psychometric properties (43) assessed various Life Skills including: Decision-Making Skills, Relaxation Skills, Communication Skills, Social Skills, Assertiveness Skills, and Conflict Resolution Skills. All Life Skills items asked respondents to indicate how much they agreed or disagreed with statements using a five-point Likert scale with responses options ranging from “Strongly Disagree” (1) to “Strongly Agree” (5). There were three items that assessed Decision-Making Skills (e.g., “I make sure I have all the information I need to make the best decision”); five items that assessed Relaxation Skills (e.g., “To cope with anxiety, I breathe in slowly, hold my breath for a count of four, and breathe out slowly”); three items that assessed Communication Skills (e.g., “I make sure that my non-verbals match what I am saying to someone”); three items that assessed Social Skills (e.g., “I ask questions to start a conversation with someone I just met”); three items that assessed Assertiveness Skills (e.g., “I would tell someone if they gave me less change (money) than I was supposed to get back after paying for something”); and three items that assessed Conflict Resolution Skills (e.g., “I would keep quiet and avoid someone if I had a conflict with them”). For each skill, a mean summary score was calculated with a possible range from 1 to 5, with higher scores representing better skills (42). An overall Life Skills score was calculated based on participants responses to all 20 items.

### Data analysis

A combination of bivariate and multivariate statistical methods was used to analyze the data including *t*-tests, chi-squares, generalized linear models (GLMs), and multilevel MIXED models using SPSS version 28 (44). Prior to testing for intervention effects, data were checked for errors, response inconsistencies, outliers, and missing data, following standardized data screening protocols. The data analysis plan included a detailed examination of pre-test equivalence of the intervention and control group participants, attrition analysis, and hypothesis testing. An intention-to-treat analysis was used that included all participants in the analyses. For outcome analyses, a series of GLM models were conducted to examine the impact of the intervention on the post-test outcomes, controlling for the pre-test score of the outcome, race/ethnicity, and gender. Including the baseline outcome score as a covariate has been found to be an efficient data analytic strategy for testing intervention effects using a two-arm randomized pre- post-test design (45). Robust estimators were specified for the GLM analyses, which relax strict assumptions about the distribution of the dependent variable. Multilevel analyses with mixed modeling were also conducted on the same set of outcomes to adjust for the potential impact of school-level clustering effects. Both the GLM and MIXED models results are presented as a robustness check, highlighting a pattern of highly similar results across both models. In addition to testing effects on substance use frequency scores, intervention effects on current (past month) substance use proportions were examined using both GLM and MIXED model approaches. One-tailed significance tests were used to determine significance levels for the analysis of intervention effects, as warranted by the unidirectional nature of hypothesized effects and the results of previous research testing this prevention approach (16).

## Results

### Sample quality

The sample consisted of 1,799 students who provided data for the pre-test assessment and 1,447 students who provided data for the pre-test and post-test assessments. A series of analyses was conducted to examine participant attrition from the pre-test to post-test, attrition by pre-test substance use status, and differential attrition across experimental conditions. Overall attrition from pre-test to post-test was 19.6%. Although not statistically significant, attrition was greater among individuals who reported pre-test substance use in the past month (18.9%) compared to non-users (15.6%). Differential attrition threats were assessed using a two-way GLM (condition × pre-test substance use) interaction to predict participation at the post-test assessment. No significant interactions were found.

To rule out the possibility that any observed intervention effects were the result of pre-existing differences between groups, intervention and control group participants in the analysis sample were compared on relevant baseline demographic and behavioral variables as shown in Table 4. There were differences between the intervention and control groups at pre-test regarding race and ethnicity. A higher proportion of the intervention group reported they were from a racial minority [vs. White, $\chi_{\left(1\right)}^{2}$ = 23.8, *p* < 0.001]. A higher proportion of the control group reported that they were Hispanic/Latino [vs. not Hispanic/Latino, $\chi_{\left(1\right)}^{2}$ = 37.3, *p* < 0.001]. There were no pre-test differences with regard to gender. Lifetime, annual, and monthly rates of substance use were equivalent across conditions at the pre-test assessment.

**Table 4:** Baseline demographics and risk behaviors.

	**Intervention**	**Control**	**All**
Sample size	622	825	1,447
Age in years: M (SD)	11.96 (0.76)	11.92 (0.92)	11.94 (0.86)
Race/ethnicity			
Racial minority (non-white)	39.5%	27.4%	32.6%
Hispanic/Latino	6.1%	16.8%	12.2%
Sex (male)	48.8%	47.9%	48.3%
Cigarette smoking (current/lifetime)	0.4%/1.8%	1.0%/1.8%	0.7%/1.8%
Alcohol use (current/lifetime)	0.9%/6.8%	0.7%/5.4%	0.8%/6.0%
Marijuana use (current/lifetime)	1.1%/1.8%	1.2%/2.0%	1.2%/1.9%
Any substance use (current/lifetime)	1.8%/7.6%	1.6%/6.3%	1.7%/6.9%

Participants were asked about race and ethnicity (Hispanic/Latino) as separate questions.

These analyses indicated that the conditions were equivalent at pre-test and that no bias was introduced into the sample due to attrition. Despite attrition between the pre-test and post-test, there was no evidence of any impact on the internal validity of the study and the ability to make valid inferences from the analysis of intervention effects.

### Substance use

A series of GLM analyses was conducted to examine intervention effects on the frequency of each substance use behavior. Findings indicated that the intervention produced significant effects on smoking, e-cigarette use, alcohol use, drunkenness, marijuana use, and the misuse (sharing) of prescription drugs. Adjusted means for substance use frequency at the post-test assessment are presented in Table 5 for each condition, after controlling for the pre-test values of each substance, race/ethnicity, and gender. The mean smoking frequency was lower in the intervention group relative to the control group [Wald $\chi_{\left(1\right)}^{2}$ = 4.12, *p* < 0.03]. The mean e-cigarette use frequency was lower in the intervention group relative to the control group [Wald $\chi_{\left(1\right)}^{2}$ = 3.45, *p* < 0.03]. The mean alcohol use frequency was lower in the intervention group relative to the control group [Wald $\chi_{\left(1\right)}^{2}$ = 2.85, *p* < 0.05]. The mean drunkenness frequency was lower in the intervention group relative to the control group [Wald $\chi_{\left(1\right)}^{2}$ = 5.93, *p* < 0.01]. In addition, the mean marijuana use frequency was lower in the intervention group relative to the control group [Wald $\chi_{\left(1\right)=}^{2}$ 4.95 *p* < 0.02]. Finally, the sharing prescription medications mean was lower in the intervention group relative to the control group [Wald $\chi_{\left(1\right)}^{2}$ = 2.73 *p* < 0.05].

**Table 5:** Adjusted means at post-test for substance use frequency by condition, GLM and MIXED models.

	**GLM**		**MIXED**	
	**Intervention**	**Control**	**Intervention**	**Control**
	**Mean (SE)**	**Mean (SE)**	**Mean (SE)**	**Mean (SE)**
Cigarette smoking	1.07 (0.02)	1.14 (0.03)*	1.08 (0.04)	1.17 (0.04)*
E-cigarette use	1.14 (0.02)	1.21 (0.03)*	1.12 (0.05)	1.26 (0.05)*
Alcohol use	1.16 (0.02)	1.23 (0.03)*	1.20 (0.04)	1.25 (0.05)
Drunkenness	1.06 (0.02)	1.15 (0.03)**	1.07 (0.03)	1.19 (0.04)**
Marijuana use	1.05 (0.01)	1.12 (0.03)*	1.10 (0.04)	1.17 (0.05)
Prescription drug misuse	1.06 (0.02)	1.12 (0.03)*	1.10 (0.04)	1.15 (0.04)

*p < 0.05, **p < 0.01.

A series of analyses was also conducted using MIXED modeling to account for the fact that students were clustered within schools. These were random intercept models with fixed effects for intervention group, race/ethnicity, and gender and controlled for pre-test substance use. These findings, shown in Table 5, indicated that the intervention produced significant effects on smoking, e-cigarette use, and drunkenness. The mean smoking frequency was lower in the intervention group relative to the control group [*F*_(1, 19.9)_ = 3.76, *p* < 0.04]. The mean e-cigarette use frequency was lower in the intervention group relative to the control group [*F*_(1, 17.5)_ = 4.02, *p* < 0.03]. The mean drunkenness frequency was lower in the intervention group relative to the control group [*F*_(1, 16.9)_ = 6.68, *p* < 0.01].

The final set of intervention analyses on substance use outcomes, shown in Table 6, examined effects on current (past month) substance use proportions using both the GLM and MIXED model approaches. The GLM analyses, shown in Table 6, revealed that the proportion of students reporting e-cigarette use was significantly lower in the intervention group relative to the control group [Wald $\chi_{\left(1\right)}^{2}$ = 3.22, *p* < 0.04]. The proportion of students reporting alcohol use was significantly lower in the intervention group relative to the control group [Wald $\chi_{\left(1\right)}^{2}$ = 4.09, *p* < 0.03]. The proportion of students reporting drunkenness was significantly lower in the intervention group relative to the control group [Wald $\chi_{\left(1\right)}^{2}$ = 7.54, *p* < 0.01]. The proportion of students reporting marijuana use was significantly lower in the intervention group relative to the control group [Wald $\chi_{\left(1\right)}^{2}$ = 5.33, *p* < 0.01]. The MIXED model analyses, shown in Table 6, revealed that the proportion of students reporting e-cigarette use was significantly lower in the intervention group relative to the control group [*F*_(1, 12.5)_ = 3.52, *p* < 0.05]. The proportion of students reporting drunkenness was significantly lower in the intervention group relative to the control group [*F*_(1, 12.5)_ = 5.26, *p* < 0.02].

**Table 6:** Adjusted proportions for current (past month) use at post-test for substance use by condition, GLM and MIXED models.

	**GLM**		**MIXED**	
	**Intervention**	**Control**	**Intervention**	**Control**
	**Proportion (SE)**	**Proportion (SE)**	**Proportion (SE)**	**Proportion (SE)**
Cigarette smoking	0.65 (0.33)	1.46 (0.46)	1.23 (0.60)	2.48 (0.64)
E-cigarette use	0.52 (0.33)	1.71 (0.51)*	0.94 (0.73)	2.86 (0.80)*
Alcohol use	0.74 (0.38)	2.31 (0.58)*	2.02 (0.83)	3.28 (0.93)
Drunkenness	0.45 (0.20)	1.79 (0.63)**	1.34 (0.63)	3.31 (0.69)*
Marijuana use	0.26 (0.14)	1.02 (0.42)**	1.96 (1.80)	2.67 (1.45)
Prescription drug misuse	0.56 (0.24)	1.18 (0.45)	1.51 (0.56)	2.14 (0.60)

*p < 0.05, **p < 0.01.

All intervention analyses included race/ethnic background and gender as covariates, which did not reach statistical significance in any analyses. Furthermore, intervention effects were conducted separately by gender, and those analyses revealed no gender-specific findings.

### Health knowledge

Using the GLM approach, the post-test Health Knowledge mean was higher in the intervention group relative to the control group [M = 10.67 vs. M = 9.58, Wald $\chi_{\left(1\right)}^{2}$ = 66.40, *p* < 0.001]. As shown in Table 7, analyses of health knowledge related to specific types of substance use showed significant intervention effects, with higher post-test means in the intervention relative to the control group for Smoking Knowledge [Wald $\chi_{\left(1\right)}^{2}$ = 107.2, *p* < 0.001], Alcohol Use Knowledge [Wald $\chi_{\left(1\right)}^{2}$ = 47.65, *p* < 0.001], Marijuana Use Knowledge [Wald $\chi_{\left(1\right)}^{2}$ = 16.77, *p* < 0.001], and Prescription Drug Misuse Knowledge [Wald $\chi_{\left(1\right)}^{2}$ = 34.03, *p* < 0.001].

**Table 7:** Adjusted means at post-test for knowledge variables by condition, GLM and MIXED models.

	**GLM**		**MIXED**	
	**Intervention**	**Control**	**Intervention**	**Control**
	**Mean (SE)**	**Mean (SE)**	**Mean (SE)**	**Mean (SE)**
Health knowledge				
Smoking/E-cigarette use	3.70 (0.04)	3.10 (0.04)***	3.49 (0.11)	2.99 (0.13)*
Alcohol use	3.09 (0.05)	2.60 (0.04)***	3.04 (0.08)	2.54 (0.09)***
Marijuana use	2.32 (0.03)	2.14 (0.03)***	2.21 (0.06)	2.01 (0.08)*
Prescription drug misuse	2.05 (0.03)	1.79 (0.03)***	2.04 (0.05)	1.79 (0.05)***
Overall health knowledge	10.67 (0.10)	9.58 (0.08)***	10.22 (0.24)	9.33 (0.29)*
Skills knowledge
Self-image	2.33 (0.03)	2.05 (0.03)***	2.33 (0.05)	2.04 (0.06)***
Decision making	1.70 (0.03)	1.61 (0.03)**	1.67 (0.05)	1.62 (0.05)
Advertising/media	2.40 (0.03)	2.04 (0.03)***	2.31 (0.08)	1.98 (0.10)**
Violence and the media	1.84 (0.04)	1.44 (0.03)***	1.77 (0.06)	1.40 (0.07)***
Coping with anxiety	2.48 (0.03)	2.01 (0.03)***	2.39 (0.07)	1.91 (0.08)***
Coping with anger	2.57 (0.03)	2.04 (0.03)***	2.53 (0.05)	1.96 (0.06)***
Communication skills	2.47 (0.03)	2.13 (0.03)***	2.35 (0.07)	2.10 (0.08)*
Social skills	2.37 (0.03)	1.90 (0.03)***	2.33 (0.06)	1.79 (0.07)***
Assertiveness	2.61 (0.03)	2.31 (0.03)***	2.52 (0.05)	2.25 (0.06)**
Conflict resolution	2.50 (0.03)	2.29 (0.03)***	2.41 (0.06)	2.27 (0.08)
Overall skills knowledge	22.51 (0.16)	19.08 (0.14)***	21.73 (0.52)	18.76 (0.65)***

*p < 0.05, **p < 0.01, ***p < 0.001.

The MIXED model approach produced similar findings, with the post-test Health Knowledge means higher in the intervention group relative to the control group [M = 10.22 vs. M = 9.33, *F*_(1, 20.3)_ = 5.54, *p* < 0.02]. As shown in Table 7, analyses of health knowledge related to specific types of substance use showed significant intervention effects, with higher post-test means in the intervention relative to the control group for Smoking/e-Cigarette Use Knowledge [*F*_(1, 20.2)_ = 8.99, *p* < 0.04], Alcohol Use Knowledge [*F*_(1, 23.5)_ = 17.99, *p* < 0.001], Marijuana Use Knowledge [*F*_(1, 20.4)_ = 4.18, *p* < 0.03], and Prescription Drug Misuse Knowledge [*F*_(1, 24.8)_ = 13.25, *p* < 0.001].

### Skills knowledge

Using the GLM approach, comparison of the intervention and control groups for knowledge regarding the life skills taught in the intervention showed significant intervention effects. Overall Skills Knowledge was higher for the intervention group than the control group [M = 22.51 vs. M = 19.08, Wald $\chi_{\left(1\right)}^{2}$ = 240.9, *p* < 0.001]. Skills knowledge was further analyzed by type, as shown in Table 7. Higher post-test knowledge means for the intervention than the control group were observed for Self-Image [Wald $\chi_{\left(1\right)}^{2}$ = 42.23, *p* < 0.001], Decision Making [Wald $\chi_{\left(1\right)}^{2}$ = 6.44, *p* < 0.01], Advertising/Media [Wald $\chi_{\left(1\right)}^{2}$ = 70.38, *p* < 0.001], Violence and the Media [Wald $\chi_{\left(1\right)}^{2}$ = 68.06, *p* < 0.001], Coping with Anxiety [Wald $\chi_{\left(1\right)}^{2}$ = 92.71, *p* < 0.001], Coping with Anger [Wald $\chi_{\left(1\right)}^{2}$ = 176.81, *p* < 0.001], Communication Skills [Wald $\chi_{\left(1\right)}^{2}$ = 59.88, *p* < 0.001], Social Skills [Wald χ^2(1)^ = 90.91, *p* < 0.001], Assertiveness [Wald $\chi_{\left(1\right)}^{2}$ = 53.09, *p* < 0.001], and Conflict Resolution [Wald $\chi_{\left(1\right)}^{2}$ = 25.58, *p* < 0.001].

The MIXED model approach produced similar findings, with Overall Skills Knowledge higher for the intervention group than the control group [M = 21.73 vs. M = 18.76, *F*_(1, 21.8)_ = 12.85, *p* < 0.001]. Skills Knowledge was further analyzed by type, as shown in Table 8. Higher post-test knowledge means for the intervention than the control group were observed for Self-Image [*F*_(1, 21.8)_ = 15.38, *p* < 0.001], Advertising/Media [*F*_(1, 21.1)_ = 7.23, *p* < 0.01], Violence and the Media [*F*_(1, 13.2)_ = 17.74, *p* < 0.001], Coping with Anxiety [*F*_(1, 23.5)_ = 22.56, *p* < 0.001], Coping with Anger [*F*_(1, 23.5)_ = 48.13, *p* < 0.001], Communication Skills [*F*_(1, 17.8)_ = 6.20, *p* < 0.05], Social Skills [*F*_(1, 19.2)_ = 33.05, *p* < 0.001], and Assertiveness [*F*_(1, 17.1)_ = 12.32, *p* < 0.01].

**Table 8:** Adjusted means at post-test for life skills variables by condition, GLM and MIXED models.

	**GLM**		**MIXED**	
	**Intervention**	**Control**	**Intervention**	**Control**
	**Mean (SE)**	**Mean (SE)**	**Mean (SE)**	**Mean (SE)**
Decision making	3.53 (0.03)	3.42 (0.02)***	3.55 (0.04)	3.39 (0.05)**
Relaxation	3.22 (0.03)	3.13 (0.02)**	3.21 (0.05)	3.05 (0.06)*
Communication	3.67 (0.03)	3.51 (0.03)***	3.64 (0.04)	3.44 (0.05)**
Social skills	3.50 (0.03)	3.36 (0.03)***	3.49 (0.05)	3.28 (0.05)***
Assertiveness	3.35 (0.03)	3.26 (0.03)*	3.34 (0.04)	3.26 (0.04)
Conflict resolution	3.43 (0.03)	3.32 (0.02)***	3.38 (0.04)	3.28 (0.05)*
Overall life skills	3.63 (0.02)	3.56 (0.01)***	3.64 (0.03)	3.49 (0.03)**

*p < 0.05, **p < 0.01, ***p < 0.001.

### Life skills

Using the GLM approach, at the post-test, the overall mean score for the skills taught in the intervention was significantly higher for the intervention group than the control group [M = 3.63 vs. M = 3.56, Wald $\chi_{\left(1\right)}^{2}$ = 13.37, *p* < 0.001]. Furthermore, the mean scores for several individual skill variables were higher for the intervention group than the control group, as shown in Table 8, including Decision Making Skills [Wald $\chi_{\left(1\right)}^{2}$ = 10.34, *p* < 0.001], Relaxation Skills [Wald $\chi_{\left(1\right)}^{2}$ = 5.59, *p* < 0.01], Communication Skills [Wald $\chi_{\left(1\right)}^{2}$ = 15.23, *p* < 0.001], Social Skills [Wald $\chi_{\left(1\right)}^{2}$ = 10.11, *p* < 0.001], Assertiveness Skills [Wald $\chi_{\left(1\right)}^{2}$ = 5.04, *p* < 0.02], and Conflict Resolution Skills [Wald χ^2(1)^ = 13.37, *p* < 0.001].

Using the MIXED approach, findings were similar, with the overall mean score for the skills taught in the intervention significantly higher for the intervention group than the control group [M = 3.64 vs. M = 3.49, *F*_(1, 17.8)_ = 11.97, *p* < 0.02]. Furthermore, the mean scores for several individual skill variables were higher for the intervention group than the control group, as shown in Table 8, including Decision Making Skills [*F*_(1, 16.6)_ = 7.80, *p* < 0.01], Relaxation Skills [*F*_(1, 21.5)_ = 4.79, *p* < 0.02], Communication Skills [*F*_(1, 15.0)_ = 11.53, *p* < 0.01], Social Skills [*F*_(1, 19.2)_ = 12.21, *p* < 0.001], and Conflict Resolution Skills [*F*_(1, 15.5)_ = 5.43, *p* < 0.02].

## Discussion

Substance abuse remains an important public health problem. Although there has been considerable progress in developing, testing, and identifying effective approaches to prevent the onset and early stages of alcohol, tobacco, and other forms of substance use, moving from science to practice by increasing the use of evidence-based programs has been difficult. A major barrier to the widespread adoption of evidence-based prevention programs intended for delivery in school settings is the amount of classroom time required for effective programs. A related challenge is that, even when adopted, school-based programs are often not implemented with fidelity, limiting their effectiveness and potential public health benefit. To overcome these barriers, innovative approaches are needed that require less class time, improve fidelity, and provide opportunities for interactive teaching activities. A hybrid digital prevention approach, if effective, would offer the potential to overcome those implementation barriers. The current study tested an adaptation of *Life Skills Training* (LST), an evidence-based prevention program designed for implementation in the classroom by teachers or other program providers, and requiring up to 18 45-min class sessions. LST was adapted and reconfigured into a hybrid digital intervention consisting of 14 brief e-learning modules (4–10 min each) and six in-person class sessions.

Students exposed to the hybrid digital version of LST reported significantly less cigarette smoking, e-cigarette use, alcohol use, marijuana use, and prescription drug misuse than students in the control group at the post-test. Additionally, students in the hybrid LST condition had higher scores on the post-test than controls on overall knowledge of the adverse consequences of substance use, as well as knowledge of a variety of personal and interpersonal skills. Finally, students who participated in the hybrid digital LST condition had increased skills related to decision-making, coping with anxiety and anger, effective communication, social skills, assertiveness, and conflict resolution.

The results of this study indicate that in addition to being effective in preventing adolescent substance use as a classroom-based program, LST is also effective when implemented in a hybrid digital format that includes e-learning modules and in-person classroom sessions. Furthermore, this study extends previous research with the LST approach by showing that it can also prevent e-cigarette use (vaping) and the misuse of prescription drugs. On a more general level, this study provides support for the effectiveness of a hybrid digital approach to prevent adolescent substance use that combines brief e-learning modules with a small number of in-person class sessions. Such an approach offers the potential for delivering program content in a time-efficient and standardized manner, while reserving class time for in-person interactive activities such as small-group discussion and skills practice. This approach also offers the potential of facilitating increased adoption and implementation fidelity of evidence-based prevention programs, along with a correspondingly greater public health benefit.

### Digital health interventions and school-based prevention

Technology is becoming more common in the classroom and there has been a shift to a more digitized classroom. Computers, tablets, and other digital devices are currently used in over 95% of K-12 classrooms (46). Web-connected computers and digital devices are likely to continue to increase among adolescents of all ages, in and out of school. Given the increased role of multimedia technology in the learning and social environments of adolescents, online e-learning formats may represent an important and innovative new media for the dissemination of substance abuse prevention programs. It is important to conduct rigorous studies that examine the various ways that digital health interventions can be used in prevention efforts.

There are several ways that digital health interventions can be used in school-based prevention efforts. Digital health interventions can replace classroom-based programs in their entirety. While stand-alone digital health interventions for substance abuse prevention offer convenience and show some evidence of efficacy (47), they fail to provide the interactivity that is critical to effective prevention efforts. Alternatively, digital health interventions can be used in conjunction with classroom-based instruction, also in a variety of ways. Digital content can complement and reinforce what students have learned in the classroom and provide opportunities for homework. Another option, consistent with the flipped or inverted classroom approach and the hybrid model tested in the current study, is that the digital health component is implemented first to teach information and skills in a time-efficient, standardized, and engaging way while the corresponding classroom sessions provide an opportunity for skills practice. As in the present study, most of the class time can be used for activities that may not be particularly well-suited for online implementation, such as small-group discussion to promote pro-health norms or guided skills training by a trained provider to promote skill acquisition.

The present study contributes to the literature by providing evidence that this hybrid or flipped intervention model can produce meaningful and significant effects when based on an existing evidence-based program. The hybrid digital model tested in the current study may overcome potential limitations of online-only digital health interventions while also addressing barriers to the adoption and implementation of classroom-based prevention programs including limited classroom time and poor implementation fidelity. A hybrid or blended digital model that uses online technology with a limited amount of classroom time can increase the feasibility of implementing evidence-based interventions and promote more widespread adoption.

The approach tested in this study has special relevance for school-based prevention in light of the COVID-19 pandemic, where schools were forced to switch from traditional in-class instruction to either a remote or a hybrid model of in-class and online course work (48). As schools return to standard in-person learning environments, it is unlikely that there will be a complete reversal of gains made with multimodal e-learning tools (48, 49). The new hybrid digital intervention tested in the present study extends the options for delivering evidence-based prevention programming and provides a flexible resource for schools and parents faced with the challenge of teaching students when classroom instruction may not be an option. Moreover, such interventions can be used in low resource settings where students might not have individual internet-enabled digital devices, but have access to school-sited computer labs.

### Implications for substance abuse prevention

The hybrid version of *Life Skills Training* tested in this study offers considerable potential as an innovative multimedia prevention resource that educators can offer to students seeking technologically sophisticated computer-based learning opportunities. At the same time, the hybrid approach maintains teacher presence and helps create an interactive atmosphere conducive to learning, which is often a concern that impedes the adoption of entirely remote e-learning programs.

Taken together, these findings hold considerable promise for the use of hybrid preventive interventions, and demonstrate that substance abuse prevention programs conducted during middle school using hybrid e-learning modules plus a limited number of classroom sessions can produce meaningful reductions in substance use and improve important life skills. This hybrid approach offers a flexible intervention delivery model that capitalizes on the strengths of both delivery modalities: an engaging self-paced e-learning format to efficiently present information and promote knowledge acquisition and in-person class time to provide opportunities for the application and practice of the knowledge and skills taught through the online e-learning program.

### Strengths and limitations

There are several strengths of the present study including a national sample, rigorous research design, standardized and well-tested protocols, and an intervention adapted from an evidence-based prevention approach. The study included schools, teachers, and students from different regions of the US to increase representativeness of the sample and promote generalizability of the findings. Standardized and well-tested protocols were used for recruiting schools, collecting data, and tracking participants over time. A rigorous randomized controlled evaluation design was used, along with confidential, standardized self-report surveys and measures with well-established psychometric properties. The intervention approach for the current study was adapted from a prevention model that has been extensively tested with results published in peer reviewed journals, and the study was based on theory and methods derived from over 30 years of research in the field of prevention science.

Limitations of the present study included the possibility of underreporting of sensitive behaviors, attrition of high-risk students, and no data on sexual minority status. Another limitation is that lower-than-expected sample sizes and rates of substance use resulted in inadequate statistical power for some mixed model analyses. The net effect of these limitations is a more conservative test of the effectiveness of this approach, making it more difficult to demonstrate program effects. Therefore, the presence of prevention effects in the face of these limitations provides strong empirical support for the effectiveness of this prevention approach.

### Future directions

Future research is needed to determine the long-term durability of this prevention approach as well as to determine whether intervention effects are moderated by the sequence of implementing e-learning and in-person program material. Research is also needed to investigate whether the delivery model tested in this study can be modified to further decrease or even eliminate the need for classroom time. An interesting line of future research might explore the effectiveness of online interactive sessions or virtual synchronous teacher-led sessions to determine if they can serve the same function and have the same impact as in-person teacher-led classroom sessions. Other related issues to explore are whether online breakout rooms could be structured to facilitate skills practice or whether a similar intervention is effective when delivered either synchronously (with instructors and students in a computer lab) or asynchronously where students complete assignments independent of in-class time. Another possible direction for future research would be to determine whether the interactivity of teacher-led, class sessions could be replicated using other technologies such as virtual reality.

## Conclusions

The current study provides strong evidence for the effectiveness of an adapted e-learning version of the LST program when combined with a small number of in-person class sessions. Findings from this study showed significant reductions in substance use and increases in health knowledge, skills knowledge, and life skills among students who received a hybrid digital preventive intervention compared to students who did not. This study also extends previous research with LST by demonstrating prevention effects on e-cigarette use and prescription drug misuse. The hybrid digital approach tested in this study is not only effective, but also holds promise for substantially reducing barriers to the uptake of evidence-based preventive interventions. Because the hybrid LST program is attractive and engaging, can be accessed using a variety of digital devices, and requires minimal class time, this prevention approach offers considerable promise for large-scale dissemination, adoption, and implementation. The results of this study further indicate that effective digital health interventions can offer the potential to transfer health information in a way that is flexible, accessible, and requires less classroom time and staffing. Providing prevention content in an online e-learning format can ensure that students receive standardized and engaging content accessed through digital devices that students are familiar with and frequently use. Finally, during a time when schools require increased flexibility and access to remote implementation modalities to address disruptions brought on by the COVID-19 pandemic and other challenges, the findings from this study may have particular public health relevance.

## Data availability statement

The raw data supporting the conclusions of this article will be made available by the authors, without undue reservation.

## Ethics statement

The studies involving human participants were reviewed and approved by the Institutional Review Board, National Health Promotion Associates. Written informed consent from the participants' legal guardian/next of kin was not required to participate in this study in accordance with the National Institutes of Health (NIH) guidance and institutional requirements.

## Author contributions

KG supervised all study activities, including study design, data collection, data analysis and interpretation, and wrote the first draft of the manuscript. CW was involved in study design, data collection, and contributed significantly to the writing of the manuscript. CB was involved in the intervention development and contributed significantly to the writing of the manuscript. SS was involved in the data management and contributed significantly to the writing of the manuscript. GB provided oversight of the study and intervention development and contributed significantly to the writing of the manuscript. All authors have read and approved the final manuscript.

## Funding

This research was funded by the National Institute on Drug Abuse, Grant Number: R44DA040358.

## Conflict of interest

Authors CW, CB, SS, and GB are employees at National Health Promotion Associates (NHPA), which markets the program adapted and tested in this project. Author KG is a former employee of NHPA and currently serves as a consultant to NHPA.

## Publisher's note

All claims expressed in this article are solely those of the authors and do not necessarily represent those of their affiliated organizations, or those of the publisher, the editors and the reviewers. Any product that may be evaluated in this article, or claim that may be made by its manufacturer, is not guaranteed or endorsed by the publisher.

## Footnotes

^1^Botvin GJ, Griffin KW, Scheier LM, Williams C. *Long-Term Behavioral Effects of a School-Based Prevention Program on Illicit Drug Use Among Young Adults*. Manuscript submitted for publication.
